# Reduced proportions of natural killer T cells are present in the relatives of lupus patients and are associated with autoimmunity

**DOI:** 10.1186/ar2505

**Published:** 2008-09-10

**Authors:** Joan Wither, Yong-chun Cai, Sooyeol Lim, Tamara McKenzie, Nicole Roslin, Jaime O Claudio, Glinda S Cooper, Thomas J Hudson, Andrew D Paterson, Celia MT Greenwood, Dafna Gladman, Janet Pope, Christian A Pineau, C Douglas Smith, John G Hanly, Christine Peschken, Gilles Boire, CaNIOS Investigators, Paul R Fortin

**Affiliations:** 1 Arthritis Centre of Excellence; Division of Genetics and Development, Toronto Western Hospital Research Institute, University Health Network; Departments of Medicine and Immunology, University of Toronto, Bathurst Street, Toronto, Ontario, M5T 2S8, Canada; 2 Toronto Western Hospital Research Institute, University Health Network, Bathurst Street, Toronto, Ontario, M5T 2S8, Canada; 3 Program in Genetics and Genome Biology, The Hospital for Sick Children Research Institute, College Street, Toronto, Ontario, M5G 1L7, Canada; 4 United States Environmental Protection Agency, Pennsylvania Avenue NW, Washington, District of Columbia 20460, USA; 5 McGill University and Genome Quebec Innovation Centre, Penfield Avenue, Montreal, Quebec, H3A 1A4, Canada; and Ontario Institute for Cancer Research, College Street, Toronto, Ontario, M5G 1L7, Canada; 6 University of Toronto Lupus Clinic, Centre for Prognosis Studies in the Rheumatic Diseases, Toronto Western Hospital, University Health Network; Department of Medicine, University of Toronto, Bathurst Street, Toronto, Ontario, M5T 2S8, Canada; 7 Division of Rheumatology, St Joseph's Health Centre, Grosvenor Street, London, Ontario, N6A 4V2, Canada; 8 Division of Rheumatology, McGill University Health Center, Cedar Avenue, Montreal, Quebec, H3G 1A4, Canada; 9 Division of Rheumatology, Ottawa Hospital, Riverside Drive, Ottawa, Ontario, K1H 829, Canada; 10 Division of Rheumatology, Department of Medicine, Queen Elizabeth II Health Sciences Centre and Dalhousie University, Summer Street, Halifax, Nova Scotia, B3H 4K4, Canada; 11 Division of Rheumatology, Department of Medicine, Faculty of Medicine, University of Manitoba, Sherbrook Street, Winnipeg, Manitoba, R3A 1M4, Canada; 12 Division of Rheumatology, Department of Medicine, Faculty of Medicine and Health Sciences, Université de Sherbrooke, 12th Avenue N, Sherbrooke, Quebec, J1H 5N4, Canada; 13 CaNIOS Investigators are listed in the Acknowledgments section; 14 Arthritis Centre of Excellence; Division of Health Care and Outcomes Research, Toronto Western Hospital Research Institute, University Health Network; Department of Medicine, University of Toronto, Bathurst Street, Toronto, Ontario, M5T 2S8, Canada

## Abstract

**Introduction:**

Systemic lupus erythematosus is a genetically complex disease. Currently, the precise allelic polymorphisms associated with this condition remain largely unidentified. In part this reflects the fact that multiple genes, each having a relatively minor effect, act in concert to produce disease. Given this complexity, analysis of subclinical phenotypes may aid in the identification of susceptibility alleles. Here, we used flow cytometry to investigate whether some of the immune abnormalities that are seen in the peripheral blood lymphocyte population of lupus patients are seen in their first-degree relatives.

**Methods:**

Peripheral blood mononuclear cells were isolated from the subjects, stained with fluorochrome-conjugated monoclonal antibodies to identify various cellular subsets, and analyzed by flow cytometry.

**Results:**

We found reduced proportions of natural killer (NK)T cells among 367 first-degree relatives of lupus patients as compared with 102 control individuals. There were also slightly increased proportions of memory B and T cells, suggesting increased chronic low-grade activation of the immune system in first-degree relatives. However, only the deficiency of NKT cells was associated with a positive anti-nuclear antibody test and clinical autoimmune disease in family members. There was a significant association between mean parental, sibling, and proband values for the proportion of NKT cells, suggesting that this is a heritable trait.

**Conclusions:**

The findings suggest that analysis of cellular phenotypes may enhance the ability to detect subclinical lupus and that genetically determined altered immunoregulation by NKT cells predisposes first-degree relatives of lupus patients to the development of autoimmunity.

## Introduction

Systemic lupus erythematosus (SLE) has a complex genetic basis, with genome-wide scans demonstrating significant or suggestive linkage between SLE and multiple chromosomal regions [1-3]. Despite the recent success of genome-wide association studies, the precise informative allelic polymorphisms contained within many of these regions remain unidentified [4,5]. This lack of knowledge reflects the facts that most linkage and association studies have investigated the association with the global phenotype of lupus, which is clinically heterogeneous, and that multiple genes act in concert to produce lupus, each having a relatively minor effect. Given this complexity, analysis of subclinical phenotypes may increase the power to detect basic pathogenic mechanisms and to define genetic susceptibility more precisely.

Murine models of lupus exhibit genetic complexity similar to that in their human counterparts [6]. However, in murine lupus study of allelic polymorphisms has been greatly aided by the ability to create congenic mice in which a single susceptibility allele, or small cluster of alleles, are back-crossed onto a normal genetic background. Notably, these congenic mice frequently exhibit subclinical phenotypes that are characterized by production of anti-nuclear antibodies (ANAs) and/or cellular changes indicative of increased B-cell or T-cell activation [7-9]. These findings suggest that the relatives of lupus patients, while lacking the full complement of genes required for development of clinical SLE, may share sufficient lupus susceptibility alleles to develop subclinical immunologic phenotypes. This concept is supported by the well documented observation that first-degree relatives of lupus patients have an increased prevalence of ANAs and other lupus-associated autoantibodies as compared with the general population [10,11], and these phenotypes have successfully been used to map genetic loci that promote production of autoantibodies in lupus patients and their family members [12,13].

Despite a relative abundance of data examining serologic phenotypes in the family members of lupus patients, relatively little is known about the cellular phenotype of these individuals. Lupus patients have a number of cellular phenotypic abnormalities, including the following: increased numbers of autoantibody secreting B cells [14,15]; increased numbers of recently activated T and B cells [16-21]; altered proportions of naïve and memory T and B cell populations [17,21-23]; and deficiencies of regulatory T-cell subsets such as natural killer (NK)T [24,25] and T-regulatory (T_reg_) cells [26-28]. Here we examined whether first-degree relatives of lupus patients share some of these distinctive cellular abnormalities.

## Materials and methods

### Subjects and data collection

All patients fulfilled four or more of the revised 1997 American College of Rheumatology criteria for the classification of SLE and had two living parents who agreed to participate in the study. In total 144 patients, 288 parents, and 79 siblings were investigated. Population control individuals for the lupus patients were obtained by random digit dialing, which permitted general matching for geographic area. Additional control individuals matching the age distribution of the parents of the lupus patients were obtained through advertisements at the University Health Network and local community centers. Control individuals with a family history of lupus were excluded from the study. The study was approved by the Research Ethics Board of the University Health Network and each participating recruitment center.

After providing an informed consent, all subjects had blood drawn for isolation of DNA, cellular analysis and serologic testing, and completed a case report questionnaire. This form included basic information on demographics, family history, lifestyle and medical history, including specific questions on autoimmune diseases, medications, and comorbidities. In addition, the physicians of patients and family members with lupus completed a questionnaire, which enabled calculation of the Systemic Lupus Erythematosus Disease Activity Index 2000 (SLEDAI-2K), a validated measure of lupus disease activity and damage.

### Cellular phenotyping

Heparinized whole peripheral blood was transported by courier overnight at room temperature, and the following day, approximately 16 to 20 hours after blood drawing, peripheral blood mononuclear cells (PBMCs) were isolated by Ficoll density gradient centrifugation. All samples were handled similarly regardless of the city of origin, and there was no difference in the time-to-analysis of samples from patients, family members, or control individuals. Isolated PBMCs were stained with various combinations of conjugated mAbs, to discriminate between cellular populations and to identify activated cells. Stained cells were fixed with 2% paraformaldehyde and analyzed by flow cytometry using a FACScalibur instrument (BD Biosciences, Missisauga, ON, Canada). The following conjugated mAbs were obtained from BD Biosciences: allophycocyanin-conjugated anti-CD3 (UHT1), anti-CD20 (2H7), anti-CD4 (RPA-T4), and anti-CD8 (RPA-T8); PE-conjugated IgG_2b _(27–35), IgG_1 _(MOPC-21), and anti-CD8 (RPA-T8), anti-CD45RO (UCHL1), anti-CD38 (H1T2), anti-CD69 (FN50), and anti-CD27 (M-T271); and FITC-conjugated IgG_1 _(MOPC-21), IgG_2a _(G155-178), and anti-CD4 (RPA-T4), anti-CD45RA (HI100), anti-CD27 (MT271), anti-CD80 (L307.4), anti-CD86 (2331 [FUN-1]), and anti-CD25 (M-A251). mAbs specific for Vα24 (C15) and Vβ11 (C21) were obtained from Immunotec (Marseille, France).

For most cellular populations 20,000 events were analyzed; however, 50,000 events were examined for enumeration of activated B cells and 200,000 lymphoid events for quantitation of NKT and T_reg _cells. The number of lymphocytes per milliliter was calculated from the number of PBMCs obtained per milliliter blood and the proportion of lymphocytes in the total cellular population, as determined by flow cytometry, acquiring all events.

**Figure 1:**
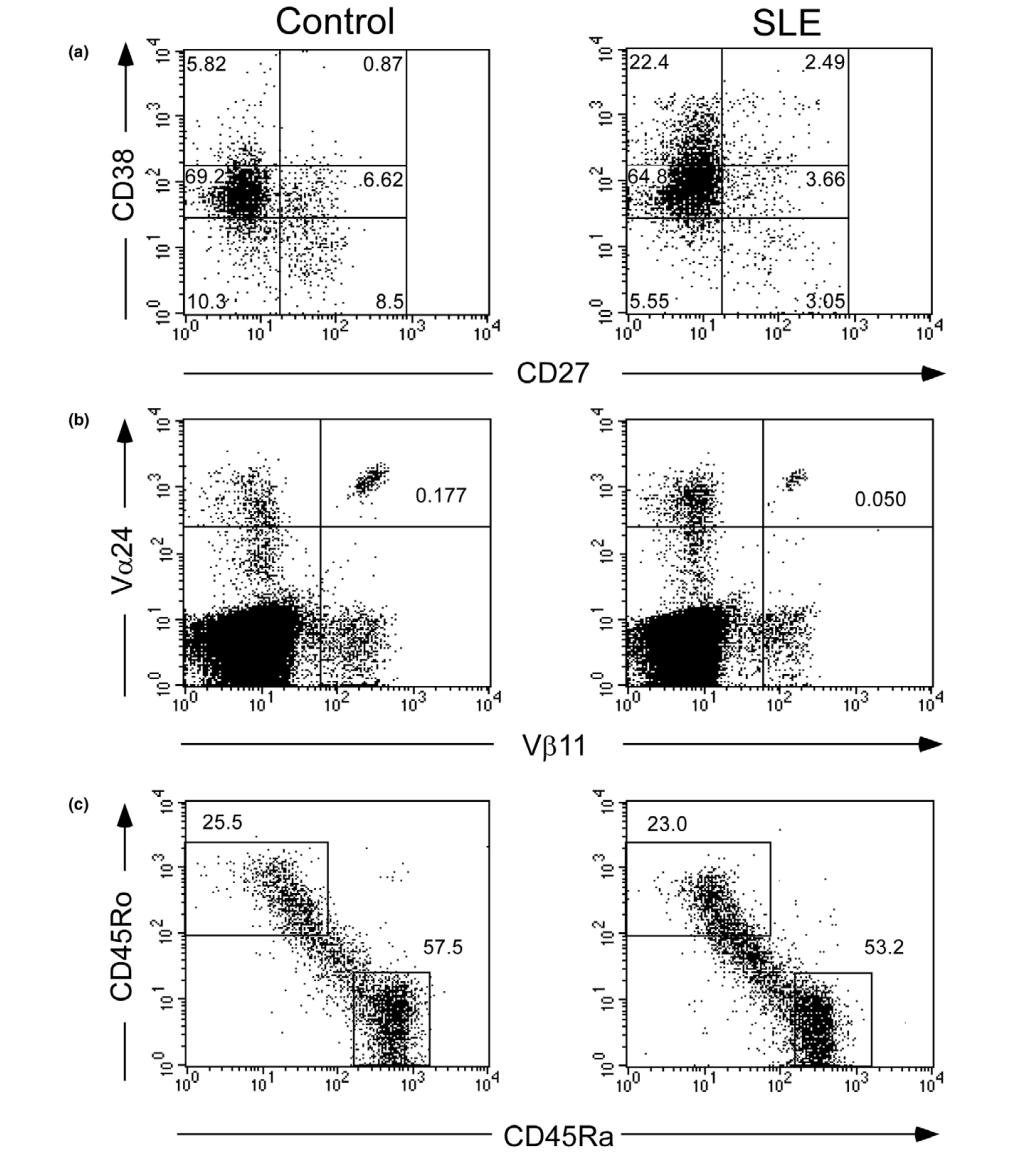
Flow cytometry profiles showing gates used to identify various lymphocyte populations. Peripheral blood mononuclear cells from representative control individuals and lupus patients were stained with combinations of conjugated mAbs, fixed, and analyzed by flow cytometry, gating on the lymphoid population as determined by forward and side staining characteristics. **(a) **Cells were stained with a combination of anti-CD20, anti-CD38, and anti-CD27 mAbs to distinguish peripheral blood B-cell subsets. Shown are dot plots, gated on CD20^+ ^cells, with four regions defined by the levels of staining with anti-CD27 and anti-CD38, as determined by staining with a relevant isotype control. Using this combination of stains, B cells can be divided into naïve transitional (CD27^-^CD38^++^) naïve mature (CD27^-^CD38^-/+^), memory (CD27^+^CD38^-/+^), and pre-germinal center (CD27^+^CD38^++^) populations. **(b) **Cells were stained with anti-CD3 in combination with anti-Vα24 and anti-Vβ11 mAbs. Shown are dot plots gated on the CD3^+ ^population. The top right quadrant represents the Vα24^+^Vβ11^+ ^invariant NKT cell population that has been proposed to play a regulatory role in autoimmunity. **(c) **Cells were stained with anti-CD4 or anti-CD8 (shown) in combination with anti-CD45RA and anti-CD45RO to identify naïve (CD45RA^+^CD45RO^-^; bottom right) and memory (CD45RA^-^CD45RO^+^; top left) cell subsets. mAb, monoclonal antibody; NK, natural killer; SLE, systemic lupus erythematosus.

For all stains, PBMCs were first gated on the lymphocyte population based on forward and side scatter characteristics. For B-cell populations, CD20^+ ^cells were gated and the results expressed as a proportion this population (Figure 1). For the B-cell activation markers CD80, CD86 and CD69, relevant populations were gated using dot plots and data from these populations plotted as a histogram. The positively staining cells were determined by comparison with an isotype control, with background isotype control staining being subtracted. The proportions of CD3^+^, CD3^+^CD4^+^, and CD3^+^CD8^+ ^cells are expressed as a percentage of the total lymphoid population. For all other T-cell phenotypes, cells have been gated on the population indicated by the first stain (for example, CD3^+^, CD4^+^, or CD8^+^) and results are expressed as a proportion of this gated population (Figure 1). Background staining with a relevant isotype control has been subtracted for the T-cell activation marker CD69. For the T_reg _cell population, the proportion of CD4^+ ^cells that were CD25^bright ^was determined using a region that was set based on CD25 staining of the CD4^- ^population, so that under 1% of the CD4^- ^population stained brightly, which permits identification of a population that is enriched for regulatory function [28].

In preliminary experiments it was determined that the delay in isolation of the PBMCs had no impact on cell number and viability (>95%), activation status, or the relative proportions of the majority of cellular populations within the lymphocyte gate in lupus patients and control individuals. However, the proportion of plasma cells within the PBMC population was significantly reduced after overnight transport. Because the majority of these cells are not contained within the lymphoid gate, the loss of this cell population had minimal impact on the proportions of the other cell populations examined.

### Serologic testing

Serum samples were screened for ANA at a 1:40 dilution using a kit with HEp-2 cell coated slides, as per the manufacturer's instructions (Antinuclear Antibody Test Kit with Stabilized Substrate, Antibodies Incorporated). Immunofluorescence was quantified using Image J1.37C software on digital images obtained with a Zeiss Axioplan 2 imaging microscope. Samples were graded based upon the percent of positive control staining above negative control staining, with a positive test being >25% above background. Anti-double-stranded DNA (dsDNA) antibody levels were determined by an in house ELISA, using calf thymus dsDNA as a substrate.

### Statistical analysis

All data were verified and double entered in an Access database. Differences for various cellular phenotypes between groups were estimated using the Wilcoxon test and using the van Elteren test, which is a rank-based Wilcoxon nonparametric test that uses weighted stratification to control for the effect of covariates [29]. Some cellular phenotypes exhibited strong deviations from normal distributions, even after log transformation; hence, the use of a nonparametric test minimizes the impact that outliers have on test statistics. Correlations between cellular phenotypes and disease activity, prednisone dose, and the levels of anti-dsDNA antibodies in the probands were determined using Spearman's rank correlation coefficient. The effect of age (stratified into <40 years, 40 to 60 years, and >60 years) and sex on the cellular variables in control individuals were determined using the Kruskal-Wallis test to assess independently the impacts of sex and age, and the Friedman rank sum test to assess the impact of age after controlling for sex and *vice versa*. Correlation of the NKT cell trait between relatives was determined using Spearman's rank correlation.

## Results

### Subject demographics

The clinical characteristics of the lupus patients are shown in Table 1. Sixty-six per cent of the patients were taking hydroxychloroquine and 40% were taking immunosuppressive drugs (23.4% azathioprine, 11.3% mycophenolate mofetil, 8.3% methotrexate, and 0.7% cyclophosphamide) at the time of the study. The mean age of the patients was 34.7 ± 9.0 (median 35.0) years, fathers 63.6 ± 9.0 (median 63.7) years, mothers 61.0 ± 8.9 (median 60.9) years, siblings 35.4 ± 9.1 (median 35.2) years, and control individuals 45.8 ± 13.0 (median 47.3) years. Eighty nine per-cent of the patients, 61% of siblings, and 82% of control individuals were female. The majority of patients were Caucasian (85.3%) with the remaining patients being Asian (7.2%), black (2.2%), Middle Eastern (1.4%), Aboriginal (0.7%), and Jewish (0.7%). Control individuals had a similar distribution of ethnic backgrounds, which was not significantly different from the probands or their parents.

**Table 1:** Demographic characteristics of 144 lupus patients

Characteristic	Value (mean ± SD)	Median	Range
ACR criteria	5.35 ± 1.29	5.00	4 to 9
Disease duration (years)	9.26 ± 6.72	7.68	0.1 to 30.3
Age at diagnosis (years)	25.44 ± 9.18	23.70	6.0 to 51.3
SLEDAI-2K score	5.61 ± 5.63	4.00	0 to 30
SLAM-2 score	5.54 ± 3.91	5.00	0 to 18
SLICC score	0.94 ± 1.20	1.00	0 to 6
Prednisone dose (mg/day)	6.50 ± 9.83	2.50	0 to 60

ACR, American College of Rheumatology; SD, standard deviation; SLAM-2, Systemic Lupus Activity Measure-2; SLEDAI-2K, Systemic Lupus Erythematosus Disease Activity Index 2000; SLICC, Systemic Lupus International Collaborating Clinics damage.

### Presence of multiple cellular abnormalities in lupus patients

Preliminary to examination of the family members of lupus patients for cellular phenotypic abnormalities, we first sought to confirm that the cellular abnormalities reported in the literature were present in our lupus population. Analysis of our lupus probands revealed a number of cellular abnormalities in comparison with control individuals (Table 2). Lupus patients had significantly increased proportions of activated B cells, as demonstrated by the increased percentage of CD20^+ ^cells with elevated levels of CD69 and increased proportion of CD86^+ ^cells in the CD27^+ ^B-cell compartment. They also had increased CD4^+ ^T-cell activation, with an increased proportion of recently activated CD69^+^CD4^+ ^T cells. Consistent with reports in the literature, lupus patients had a relative decrease in mature naïve cells and increase in transitional and pre-germinal center cells in their B-cell compartment [21-23]. However, we did not find increased proportions of memory CD4^+ ^or CD8^+ ^T cells in our patients. Furthermore, contrary to previous reports demonstrating decreased proportions of CD4^+^CD25^+ ^T_reg _cells in lupus, we did not observe any alterations in this population. In contrast, lupus patients had markedly decreased proportions of NKT cells, as identified by analysis of CD3^+^Vα24^+^Vβ11^+ ^cells, which have been shown to correlate strongly with the invariant CD1d-restricted NKT cell population that is proposed to play an inhibitory role in autoimmune disease [30-32].

**Table 2:** Cellular phenotypes of lupus probands, first-degree relatives and controls

Cell population gated	Cell types	Probands (n = 144)	First-degree relatives (n = 357)	Controls (n = 102)
Lymphocytes (× 10^6^/ml)		0.63 ± 0.31*** (<0.0001)	0.83 ± 0.32	0.83 ± 0.37
CD20^+^	B cells	14.54 ± 9.12	15.19 ± 7.10 (0.014)	13.84 ± 4.95
CD20^+^CD27^-^CD38^-/+^	Naïve mature B cells	60.59 ± 16.54*	60.24 ± 15.06** (0.0051)	65.47 ± 11.65
CD20^+^CD27^-^CD38^++^	Transitional B cells	14.63 ± 11.61* (0.009)	10.58 ± 8.04	10.11 ± 5.92
CD20^+^CD27^-^CD80^+^	Activated naïve B cells	2.75 ± 4.72	1.50 ± 2.37	2.21 ± 5.51
CD20^+^CD27^-^CD86^+^	Activated naïve B cells	6.93 ± 9.43	4.04 ± 5.64*** (0.0009)	6.44 ± 7.19
CD20^+^CD27^+^CD38^-/+^	Memory B cells	23.40 ± 15.79	28.15 ± 15.67*	24.32 ± 11.50
CD20^+^CD27^+^CD38^++^	Pre-germinal center B cells	2.77 ± 2.98*** (0.003)	1.49 ± 1.91	1.68 ± 3.06
CD20^+^CD27^+^CD80^+^	Activated memory/pre-germinal center B cells	18.91 ± 11.69*	16.59 ± 9.53	15.84 ± 8.36
CD20^+^CD27^+^CD86^+^	Activated memory/pre-germinal center B cells	12.12 ± 9.64*** (0.0039)	8.10 ± 6.52	9.00 ± 8.47
CD20^+^CD69^+^	Recently activated B cells	20.40 ± 15.84*** (<0.0001)	13.05 ± 12.00	10.85 ± 8.93
CD3^+^	T cells	63.44 ± 14.68	61.23 ± 12.68*	64.8 ± 9.70
CD3^+^CD4^+^	CD4^+ ^T cells	34.50 ± 12.77*** (0.0001)	40.06 ± 11.91	40.77 ± 9.98
CD3^+^CD8^+^	CD8^+ ^T cells	25.24 ± 11.01*	19.38 ± 9.30*	21.18 ± 7.46
CD3^+^Vα24^+^Vβ11^+^	NKT cells	0.06 ± 0.13*** (<0.0001)	0.08 ± 0.20*** (0.013)	0.11 ± 0.17
CD4^+^CD25^+^	T_reg _cells	6.62 ± 4.30	6.39 ± 3.41	6.46 ± 2.98
CD4^+^CD45RA^+^CD45RO^-^	Naive CD4^+ ^cells	31.62 ± 14.41	25.06 ± 13.13**	29.05 ± 12.69
CD4^+^CD45RA^-^CD45RO^+^	Memory CD4^+ ^cells	37.10 ± 14.04*	**41.63 ± 15.16 **(0.0433)	40.27 ± 11.95
CD4^+^CD69^+^	Recently activated CD4^+ ^cells	10.55 ± 11.32*** (0.0001)	7.08 ± 8.56	5.95 ± 5.92
CD8^+^CD45RA^+^CD45RO^-^	Naive CD8^+ ^cells	62.36 ± 16.37*	53.92 ± 15.15**	58.61 ± 13.53
CD8^+^CD45RA^-^CD45RO^+^	Memory CD8^+ ^cells	15.75 ± 9.91*** (0.0027)	21.35 ± 11.09	20.29 ± 10.37

Cellular phenotypes were determined by flow cytometry following staining with relevant conjugated monoclonal antibodies, as described in the Materials and methods section. Shown is the mean ± standard deviation for each group. Asterisks indicate significance as compared to controls using the Wilcoxon Test: *P < 0.05, **P < 0.005, and ***P < 0.0005. Bold numbers denote significant differences (*P *< 0.05) from control individuals using a Van Elteren test, where sex and age are covariates. The *P *values for significant differences are shown in parentheses. NK, natural killer; T_reg_, T regulatory (cell).

With the exception of the number of lymphocytes per milliliter (*P *= 0.0026), there was no significant correlation between the SLEDAI-2K and any of the cellular abnormalities examined. However, there was a significant correlation between prednisone dose or use of cytotoxic medications and several of the cellular phenotypes examined. An increased dose of prednisone was negatively correlated with the number of lymphocytes per milliliter (*P *< 0.0001) and the proportion of total B cells (*P *= 0.0007), transitional B cells (*P *= 0.0033) and CD4^+ ^T cells (*P *= 0.0003), and positively correlated with the proportion of CD8^+ ^T cells (*P *= 0.016), memory B cells (*P *= 0.034) and CD80^+ ^(*P *= 0.033) or CD86^+ ^(*P *= 0.0003) naïve B cells. In association with use of any cytotoxic drug, similar trends were observed for the proportion of total B cells (*P *< 0.0001), transitional B cells (*P *= 0.041), memory B cells (*P *= 0.0002), CD8^+ ^T cells (*P *= 0.0038), and CD80^+ ^(*P *= 0.0003) or CD86^+ ^(*P *= 0.010) naïve B cells. In addition, use of cytotoxic drugs was associated with a reduced proportion of mature naïve B cells (*P *= 0.0020) and increased proportion of pre-germinal center cells (*P *= 0.021). In general, anti-malarial drug use was not associated with differences in proportions of the cellular populations. Notably, the majority of the cellular phenotypes that exhibited strong statistical differences between control individuals and probands did not vary with drug therapy or varied in a way that could not account for the differences observed.

Because our populations contained individuals of both sexes and with a broad age range, we questioned whether any of the cellular phenotypes varied with age or sex within our control population. Using a multivariate analysis incorporating age and sex, there was a significant correlation between increased age and an increased proportion of memory (CD45RA^-^RO^+^; *P *= 0.042) CD4^+ ^cells and decreased proportions of CD3^+ ^T cells (*P *= 0.002), CD8^+ ^T cells (*P *= 0.003), and naïve CD4^+ ^cells (CD45RA^+^RO^-^; *P *= 0.0007). Males had significantly reduced proportions of activated B cells (CD27^-^CD86^+^, *P *= 0.004; CD27^+^CD80^+^, *P *= 0.019; and CD27^+^CD86^+^, *P *= 0.0002) together with increased proportions of CD8^+ ^T cells (*P *= 0.041). We therefore extended our statistical evaluation to control for these two covariates in all subsequent analyses where comparisons were being made between family groups and control individuals. As shown in Table 2, for all of the phenotypic differences that were significant at the *P *< 0.005 level between lupus patients and control individuals using the Wilcoxon test; strong statistical significance (*P *< 0.005) was retained when the van Elteren test (see Materials and methods, above) was used to take these covariates into account.

### Cellular abnormalities in the family members of lupus patients

**Figure 2:**
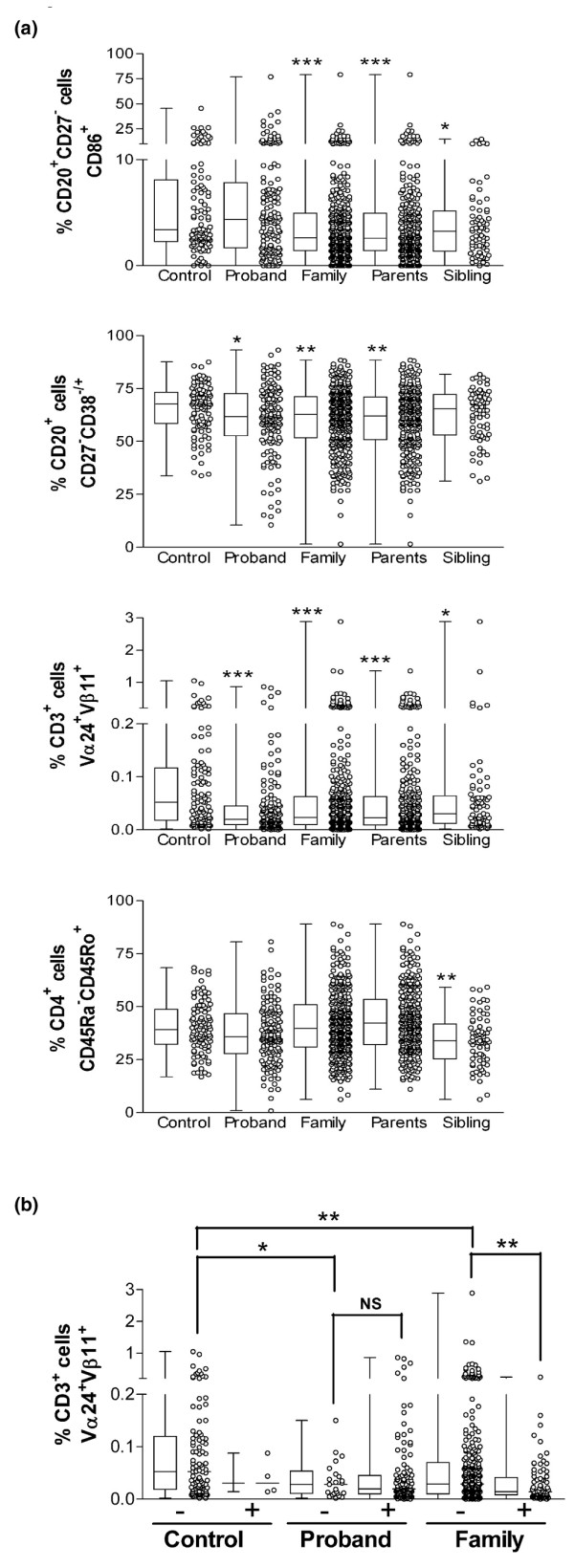
Scatter plots for cell populations that demonstrated significant differences between first-degree relatives and control individuals. Peripheral blood mononuclear cells were stained with various combinations of conjugated mAbs, fixed, and analyzed by flow cytometry (as outlined in the Materials and methods section and shown in Figure 1). **(a) **Shown are plots for the proportion of activated naïve B cells (CD20^+^CD27^- ^cells that were CD86^+^), the proportion of B cells (CD20^+^) that had a mature naïve phenotype (CD27^-^CD38^-/+^), the proportion of NKT cells (CD3^+ ^cells that were Vα24^+^Vβ11^+^), and the proportion of memory CD4^+ ^T cells (CD45RA^-^CD45RO^+^). Results shown are for 144 (143 for NKT cells) lupus probands, 356 family (parents and siblings) members (355 for NKT cells), 287 parents (286 for NKT cells), 69 siblings, and 102 control individuals. **(b) **The proportion of NKT cells in controls, probands, and family members, stratified for the presence or absence of positive ANA status. Significant differences (*P < 0.05, **P < 0.005, and ***P < 0.0005) were determined using the Wilcoxon test. In panel a differences are as compared with control individuals, and in panel b comparisons are between indicated populations. mAb, monoclonal antibody; NK, natural killer.

We next examined whether the family members of lupus patients shared any of the cellular abnormalities that we had observed in the lupus patients. As shown in Table 2, despite previous reports in the literature indicating increased autoantibody production in the relatives of lupus patients [10,11], the proportions of activated B cells, as determined by expression levels of CD69, CD80, and CD86, were not increased in the family members of our lupus patients. Indeed, there was a highly significant reduction in the proportion of CD86^+ ^naïve B cells in the family members of lupus patients as compared with control individuals. In addition, lupus family members had a significantly decreased proportion of mature naïve B cells, with a trend toward an increased proportion of memory B cells, raising the possibility that there is a low-grade increase in B-cell activation as compared with control individuals. As shown in Figure 2, consistent trends toward decreased proportions of mature naïve and CD86^+ ^naïve B cells were seen when the first-degree relatives of lupus patients were segregated into parents and siblings, but these were less pronounced in the siblings.

Although the majority of T-cell subsets examined were not different between the family members of lupus patients and control individuals, a decrease in the proportion of NKT cells was seen in the first-degree relatives of lupus patients compared with control individuals. Although the reduced proportion of NKT cells was not as pronounced as that seen in the probands (*P *= 0.0009 for relatives as compared with probands), it achieved statistical significance for both parent and sibling subpopulations when compared with control individuals (Figure 2). An increased proportion of CD4^+ ^memory T cells was also seen in the first-degree relatives as a whole, but this difference was not consistent when the parents and siblings were analyzed separately. Notably, there was no correlation between the proportion of NKT cells and the proportions of CD86^+ ^naïve B cells, mature naïve B cells, or memory CD4^+ ^T cells in the lupus family members.

### The reduced proportion of NKT cells in the family members of lupus patients correlates with the presence of a positive ANA

To determine whether the presence of positive ANA status was correlated with any of the cellular phenotypes identified in the relatives of our lupus patients, we measured IgG ANAs, using HEp-2 cells as a substrate. The frequency of ANA positive status at the time of study in our lupus patients was 85.2%, as compared with a rate of 4% ANA positivity in the control individuals (*P *< 0.001, Fisher's exact test). Consistent with previous reports, the first-degree relatives of lupus patients had a marked increase in the frequency of ANA positivity as compared with control individuals. Overall, 21.7% of family members were ANA^+ ^(*P *< 0.001 versus control individuals), with a frequency of 23.9% in the mothers (*P *< 0.001), 22.4% in the fathers (*P *< 0.001), and 16% in the siblings (*P *= 0.008). Comparison of cellular phenotypes between ANA^+ ^and ANA^- ^relatives using the van Elteren test, with age and sex as covariates, revealed that only the proportion of NKT cells was correlated with a positive ANA status (*P *= 0.009); in family members the median proportion of NKT cells was significantly lower in individuals with a positive ANA (mean ± standard deviation = 0.032 ± 0.042, median = 0.014) as compared with those who were ANA negative (mean ± standard deviation = 0.086 ± 0.23, median = 0.029).

Because very few of the first-degree relatives had elevated levels of anti-dsDNA antibodies, the association between the presence of these autoantibodies and the proportion of NKT cells was not examined. However, there was no correlation between anti-dsDNA antibody levels and the proportion of NKT cells in the probands.

### Association between autoimmune disease in the family members of lupus patients and a reduced proportion of NKT cells

A reduced proportion of NKT cells has been reported in multiple autoimmune diseases and has been noted in family members of patients with type 1 diabetes mellitus (DM) [33]. We therefore addressed whether the family members of our lupus patients had an increased frequency of autoimmune disease and investigated whether this was associated with a reduced proportion of NKT cells. As shown in Table 3, the frequency of any autoimmune disease in our control individuals was approximately 5%, which is consistent with previous population surveys [34]. The percentage of lupus patients' family members reporting any autoimmune disease was 28.3%, which was significantly increased as compared with population control individuals, with the most commonly reported autoimmune diseases being rheumatoid arthritis (11.4%), closely followed by hypothyroidism (11.2%). Although 31 relatives self-reported DM, only one mother self-reported a clinical picture consistent with type 1 DM, but this diagnosis could not be confirmed.

**Table 3:** Prevalence of self-reported autoimmune disease in the family members of lupus patients

Autoimmune disease	Father (n = 144; n [%])	Mother (n = 144; n [%])	Sibling (n = 79; n [%])	Controls (n = 102; n [%])
Autoimmune disease: any	31 (21.53)	60 (41.67)	13 (16.67)	5 (4.90)
SLE	2 (1.40)	3 (2.08)	3 (3.85)	0
Rheumatoid arthritis	14 (9.72)	26 (18.06)	2 (2.56)	2 (1.96)
Scleroderma	1 (0.69)	1 (0.70)	0	0
Dermatomyositis/polymyositis	0	2 (1.39)	0	0
Sjögrens syndrome	1 (0.69)	3 (2.08)	0	0
Antiphospholipid syndrome	0	2 (1.39)	1 (1.28)	0
Hemolytic anemia	6 (4.17)	3 (2.08)	1 (1.28)	0
Multiple sclerosis	1 (0.69)	2 (1.39)	1 (1.28)	0
Vitiligo	2 (1.39)	3 (2.08)	1 (1.28)	0
Hyperthyroid	4 (2.78)	7 (4.90)	4 (5.13)	1 (0.99)
Hypothyroid	5 (3.47)	30 (20.83)	6 (7.69)	4 (3.92)

SLE, systemic lupus erythematosus.

Every effort was made to confirm the presence of self-reported autoimmune disease, but only about 25% of autoimmune disease diagnoses could be confirmed at the time of analysis because of limitations on access to medical records. Of the 109 lupus relatives with at least one self-reported autoimmune disease, additional clinical information was available for 47, and 25 of these were confirmed positive. Because of the variability in confirmation of reported autoimmunity, cellular phenotypes were examined for both self-reported and confirmed autoimmune disease. For self-reported autoimmune disease, the presence of any autoimmune disease was associated with a significantly reduced number of lymphocytes per milliliter, reduced proportion of NKT cells, and increased proportion of CD69^+ ^B cells in comparison with control individuals (*P *= 0.022, 0.0001 and 0.041, respectively, Wilcoxon test). When these data were adjusted for age and sex, using the van Elteren test, the differences in the number of lymphocytes per milliliter and proportion of NKT cells remained significant (*P *= 0.039 and 0.0006, respectively).

To examine the association with confirmed autoimmune disease, first-degree relatives with self-reported autoimmune disease for whom additional clinical information could not be obtained were removed from the analysis, and those who did not report an autoimmune disease or whose self-reported autoimmune disease was confirmed to be absent by medical records were considered to lack autoimmune disease. Only the reduced proportion of NKT cells was significantly associated with confirmed autoimmune disease (*P *= 0.006, using the Wilcoxon test), and this remained significant after adjustment for age and sex (*P *= 0.011, using the van Elteren test).

### The reduced proportion of NKT cells in the first-degree relatives of lupus patients is independently associated with a positive ANA and autoimmune disease

The presence of a positive ANA status in the family members of lupus patients was significantly correlated with both self-reported and confirmed autoimmune disease (*P *= 0.002 and < 0.001, respectively, by Fisher's exact test). We therefore examined whether autoimmune disease and positive ANA status were independently associated with a reduced proportion of NKT cells. To address this possibility, the van Elteren test was used to control for the presence or absence of positive ANA status in the autoimmune disease analysis and *vice versa*. For both self-reported and confirmed autoimmune disease, there was a significant reduction in NKT cells when the data were controlled for ANA status (*P *= 0.0004 and 0.0032, respectively). Similarly, positive ANA status wasindependently associated with a reduced proportion of NKT cells, when the presence or absence of self-reported or confirmed autoimmune disease was taken into account (self-reported, *P *= 0.0077; confirmed, *P *= 0.0032). As illustrated in Table 4, positive ANA status and autoimmune disease were independently and cumulatively associated with a reduced proportion of NKT cells. Nevertheless, the proportion of NKT cells was reduced as compared with normal control individuals, even in first-degree relatives who were ANA negative and did not have a self-reported or confirmed autoimmune disease (*P *= 0.015 and 0.009 for self-reported and confirmed autoimmune disease, respectively, using the Wilcoxon test).

**Table 4:** Proportion of NKT cells in first-degree relatives of lupus patients, stratified by the presence of autoimmune disease and ANAs

		Self-reported autoimmune disease		Confirmed autoimmune disease	
		No	Yes	No	Yes
ANA status	Negative	0.089 ± 0.237 (0.030)	0.079 ± 0.198 (0.017)	0.092 ± 0.234 (0.035)	0.039 ± 0.054 (0.016)
	Positive	0.040 ± 0.048 (0.022)	0.020 ± 0.029 (0.009)	0.036 ± 0.046 (0.019)	0.016 ± 0.029 (0.008)

Shown is the proportion of natural killer (NK)T cells mean ± standard deviation (median) for each group. ANA, anti-nuclear antibody.

### The proportion of NKT cells is a heritable trait

To determine whether the proportion of NKT cells is genetically determined, we examined the correlation between the proportions of NKT cells between individuals within the same family. There was a significant correlation between the mid-parental value for the proportion of NKT cells and their proband's value (*r *= 0.223, *P *= 0.0079) as well as between the mid-parental value and their unaffected offspring's value (*r *= 0.416, *P *= 0.00093). A similar association was found between probands and their siblings (*r *= 0.280, *P *= 0.030).

## Discussion

In this study, most of the distinctive cellular abnormalities in lupus patients were not observed in their family members. Nevertheless, the first-degree relatives of lupus patients had reduced proportions of NKT cells and a relative shift toward increased proportions of memory and reduced proportions of naïve B and CD4^+ ^T cells, as compared with population control individuals.

Although our study is not the first to examine cellular phenotypes in first-degree relatives of lupus patients, it is the first to perform such a comprehensive examination of the multiple different cellular phenotypic abnormalities in SLE. Previous studies seeking cellular abnormalities in the family members of lupus patients focused on a limited number of phenotypes, including examination of antibody-secreting cells, NK cells, and CD56^+ ^T cells, and had significantly smaller sample sizes. Clark and coworkers [35] examined antibody-secreting cells in 25 first-degree relatives of lupus patients and found similar levels to those in control individuals. Similarly, there were no significant differences in the proportion or killing activity of NK cells between first-degree relatives of lupus patients and control individuals [36]. The proportion of CD56^+ ^T cells was also comparable in 45 first-degree relatives and control individuals [37]. Although the authors argued that this indicates that NKT cells are not reduced in the relatives of lupus patients, studies indicate that CD56 is a poor marker for the immunoregulatory invariant NKT cell population because it is also expressed on some other peripheral blood T cells [30], whereas the Vα24^+^Vβ11^+^CD3^+ ^cells examined in the present study correlate strongly with this population [30-32]. Indeed, a recent study [38] found no significant difference between the proportion of cells detected by anti-Vα24 and anti-Vβ11 staining, and those observed after staining with CD1d tetramers loaded with the α-galactosylceramide analog PBS57 or 6B11 (a mAb that recognizes the conserved region of the canonical Vα24Jα18 T cell receptor in invariant NKT cells).

Although a large number of cellular variables were assessed in this study, several findings suggest that the statistically significant differences observed between the first-degree relatives and control individuals did not occur by chance alone. In a study of 49 additional trios recruited after this study, the proportion of NKT cells in the first-degree relatives was similarly and significantly reduced as compared with control individuals (% NKT = 0.074 ± 0.12; *P *= 0.016 versus control individuals). Furthermore, the observation that the reduced proportion of NKT cells in first-degree relatives is independently and additively associated with positive ANA status and autoimmune disease strongly suggests that this reduction is of immunopathogenic and not just statistical relevance. Despite less striking differences in the proportions of memory and/or naïve B and CD4^+ ^T cells, these changes may also be of pathogenic importance. We recently showed that a nonsynonymous single nucleotide polymorphism in the SLAM molecule Ly9 is linked to development of lupus in our collection of trios [39]. We further demonstrated that this polymorphism, which is predicted to alter downstream signaling events, is associated with skewing of T-cell populations away from a naïve and toward a memory phenotype in the parents of our lupus patients.

NKT cells are a unique T-cell lineage that recognize glycolipid antigens within the context of CD1d, a nonclassical major histocompatibility complex (MHC) class I molecule. Upon activation, these cells are potent producers of immunoregulatory cytokines [31,32]. Reduced proportions of these cells have been described in a number of human autoimmune conditions, including SLE [24,25,40], scleroderma [24,40,41], Sjögren's syndrome [24,40], rheumatoid arthritis [24,40,42], multiple sclerosis [24,40,43], and type 1 DM [33,44]. In several animal models of autoimmune disease, including the nonobese diabetic model of type 1 DM [45-48], experimental autoimmune encephalomyelitis[49,50], and collagen-induced arthritis [51], deficiencies in NKT cells exacerbate disease whereas expansion and/or activation of NKT cells ameliorate disease. Results in murine models of lupus have been more conflicting, with both reduced and increased proportions of NKT cells proposed to exacerbate disease [52-56]. These disparities appear to arise, at least in part, from variations in the cytokines that are secreted by the NKT cells in the different lupus mouse models, with interleukin-4-secreting NKT cells inhibiting lupus and interferon-γ-secreting NKT cells exacerbating lupus [53,54,56,57]. Similar findings have been observed in other autoimmune mouse models [45,46,49,51,58-60], suggesting that the immune mechanisms through which NKT cells act to suppress lupus and other autoimmune diseases are similar. This concept is further strengthened by our demonstration in this study that there is an association between reduced proportions of NKT cells and diverse autoimmune diseases in first-degree relatives of lupus patients.

Although deficiencies in NKT cells have been shown to be genetically linked or to precede the development of autoimmunity in murine models of autoimmune disease, data addressing these issues in humans are sparse. In type 1 DM, reduced proportions of NKT cells were observed in high-risk relatives with anti-pancreatic autoantibodies, suggesting that NKT cell deficiencies in this disease predate the development of clinical diabetes [33]. Only a single study [25] has investigated the association between the proportion of NKT cells and disease activity. In this study, a subset of NKT cells, the CD4^-^CD8^-^Vα24JαQ expressing population, was examined, and the proportion of these cells was decreased only in active disease. In our study, we found no correlation between the proportion of total NKT cells and disease activity or drug therapy in our lupus patients, which suggests that the reduction in NKT cells in these patients does not arise as a secondary phenomenon in response to active disease or its treatment. Although first-degree relatives with positive ANA status and autoimmune disease had the lowest levels of NKT cells, significantly reduced proportions of NKT cells were still observed in family members without any clinical evidence of autoimmune disease or positive ANA status. This observation, together with the observation that the levels of NKT cells are significantly correlated between genetically related individuals within the same family, suggests that the reduced proportion of NKT cells is a heritable trait. These findings raise the possibility that one of the explanations for the clustering of multiple autoimmune disorders within the families of lupus patients is the presence of genetic polymorphisms that dictate NKT cell numbers and function, and that these changes precede the development of disease.

Aside from the changes in NKT cell numbers and the proportions of memory and/or naïve B and CD4^+ ^T cells, first-degree relatives did not generally share the same immune abnormalities as the lupus probands. In particular, the marked B-cell activation phenotype that is characteristic of lupus was absent. We previously showed that the increased B-cell activation demonstrates only a weak correlation with disease activity and is present both in newly diagnosed, untreated lupus and clinically inactivate lupus (SLEDAI-2K = 0) [21]. The findings in this report confirm these observations and demonstrate that development of positive ANA status in the relatives of lupus patients need not be associated with any markers of increased B-cell activation, suggesting that this marked B-cell activation phenotype develops after the immunologic events that lead to overt lupus. Although the levels of costimulatory molecules on B cells in the relatives of lupus patients were somewhat reduced in this study as compared with control individuals, and this achieved strong statistical significance in the case of CD86 expression in the CD27^- ^naïve B-cell population, we were unable to replicate these findings in a subsequent study of 49 trios. This observation suggests that these differences are not biologically relevant and may have resulted from undefined covariants in our populations and/or experimental methods.

## Conclusion

The abnormal B-cell and T-cell activation phenotype that is observed in lupus patients is not seen in their family members, suggesting that these abnormalities develop after the immunologic events that lead to overt lupus. However, significant genetically determined reductions in the numbers of NKT cells were observed in the first-degree relatives of lupus patients that correlate with serological and clinical autoimmunity, suggesting that altered immunoregulation by NKT cells may predispose these individuals to autoimmunity. Subtle changes were also observed in the relative proportions of naïve and/or memory B-cell and T-cell populations, and in a recent study these T cells changes were associated with a single nucleotide polymorphism in Ly9, which was linked to lupus [39]. Thus, the analysis of cellular phenotypes in the relatives of lupus patients may reveal extremely useful subclinical phenotypes to increase the power of genetic linkage studies, not only for lupus but also for other autoimmune diseases, as well as providing important clues to the genesis of these conditions.

## Abbreviations

ANA: anti-nuclear antibody; DM: diabetes mellitus; dsDNA: double-stranded DNA; NK: natural killer; mAb: monoclonal antibody; PBMC: peripheral blood mononuclear cell; SLE: systemic lupus erythematosus; SLEDAI-2K: Systemic Lupus Erythematosus Disease Activity Index 2000; T_reg_: T-regulatory.

## Competing interests

The authors declare that they have no competing interests.

## Authors' contributions

JW participated in study design; coordinated the flow cytometry, serologic studies, and data acquisition; participated in the data analysis; and drafted the manuscript. YC performed the flow cytometry, serologic studies, and data acquisition. SL, NR, CMTG, and ADP performed the statistical analyses. TM and JOC coordinated the acquisition of laboratory samples, clinical data acquisition, and entry of clinical and laboratory variables into the database. TJH, CMTG, and GSC participated in study design. DG, JP, CAP, CDS, JGH, CP, and GB participated in recruitment of patients and their family members, and acquisition of clinical data and laboratory samples. PRF participated in study design, and coordinated acquisition of clinical data and laboratory samples, as well as entry of clinical and laboratory variables into the database. All authors participated in revision of manuscript drafts and read and approved the final manuscript.
